# What do patents tell us about microalgae in agriculture?

**DOI:** 10.1186/s13568-021-01315-4

**Published:** 2021-11-23

**Authors:** Mayara Mari Murata, Luiz Rodrigo Ito Morioka, Josemeyre Bonifacio Da Silva Marques, Alessandra Bosso, Hélio Hiroshi Suguimoto

**Affiliations:** Universidade Pitágoras-Unopar, Programa de Pós-Graduação Stricto Sensu em Ciência e Tecnologia de Leite e Derivados, Londrina, Paraná Brazil

**Keywords:** Intellectual property, Database, Sustainable agriculture, Microalgae

## Abstract

Microalgae have been used widely as a biological source for several industries, such as biofuel, pharmaceutical and food. Recently, the agricultural industry has also began using microalgae as an alternative source for sustainable products to replace agrochemicals. Due to the lack of scientific articles in this research area, the objective of this study was to search for applications of microalgae and to characterize its use in agriculture using the patent documents available in three patent databases, World Intellectual Property Organization (WIPO), European Patent Office (EPO) and Brazilian Institute of Industrial Property (INPI). The search was carried out using the keyword “microalgae” and applying the filter for International Patent Classification (IPC) code “A01N” which corresponds to patents related to agriculture and cultivation of microalgae. Our patent database search returned 669 documents and 132 patents were selected for the study based on their abstracts. The first patent was registered in 1982 and described the use of microalgae *Chlorella* extract as a plant growth promoter. After that, no patent was registered for 15 years. From 2005 to 2014, only seven patents were found. However, the scenario changed from 2015 when the number of patents increased mainly in the United States, China and Europe. The patent analysis showed several applications for microalgae in the agricultural sector, such as plant growth promotion, biofertilization, plant disease control, weed management, and post-harvest quality. This review confirmed the increasing interest in microalgae-derived products in agriculture and the value of using patent documents to assess innovative areas.

## Introduction

Microalgae are a group of photosynthetic organisms consisting of eukaryotic organisms and prokaryotic cyanobacteria (blue-green algae), which have great potential as bioresource alternatives for various industries (Renuka et al. 2018). According to the Patent Landscape Report on Microalgae-Related Technologies, the three main applications for microalgae-derived products were health, energy and human nutrition, including biofuels, pharmaceutical products and food (WIPO 2020b). Microalgal research has increased in academic and private settings due to their versatile nature, high photosynthetic activity, heterotrophic growth, adaptability to domestic and industrial wastewater, simple unicellular structure and their likely ability to yield valuable co-products (Chiaiese et al. 2018).

Increasing agricultural production while preserving the environment has been a constant challenge for scientific research, as agricultural production systems need to meet the growing demand for food with the introduction of sustainable technologies (Mógor et al. 2019; Chiaiese et al. 2018) that demand natural inputs to the detriment of synthetics. In addition, the agroindustry generates large amounts of waste that can cause serious environmental problems if discarded incorrectly (Pereira et al. 2021; Bosso et al. 2020).

To overcome these challenges, microalgal applications are gaining interest in agriculture since waste generated by the agroindustry showed great potential for microalgal mixotrophic cultivation (Pereira et al. 2021). Several studies have been carried out to evaluate the efficiency of microalgal biomass production under different agro-industrial waste, such as cheese whey (Bosso et al. 2020; Patel et al. 2020) and desalination concentrate wastewater (Matos et al. 2018). Microalgae are also expanding in agriculture due to the increased use of sustainable products such as biopesticides and biofertilizers that are being introduced into the market to replace agrochemicals, which are detrimental to the environment (Guo et al. 2020). Microalgae can biosynthesize several metabolites with potential biological control of insects and phytopathogens (Costa et al. 2019) and can bring benefits to the crop such as plant growth and yield, due to their ability to enrich the soil with nutrients and enhance the utilization of macro and micronutrients as a result of soil microbial activity stimulation (Renuka et al. 2018).

Several cyanobacteria are known as an effective biofertilizer due to biological nitrogen fixation. They can fix atmospheric nitrogen (N2) by free living or symbiotic associations (Singh et al. 2016, 2019) and their biomass has been produced successfully in wastewater waste, open ponds and closed photobioreactors. The biomass has been used in agricultural crops as a slow-release bio-fertilizer (Kumar et al. 2017). In addition to soil fertility improvement, microalgae can also produce plant growth hormones, polysaccharides and antimicrobial compounds and metabolites that contribute to plant growth (Renuka et al. 2018; Guo et al. 2020). Even though microalgae produce several macromolecules that are active on plants and bring benefits to their development, their applications in crop science are still at an early stage (Carney et al. 2015; Chiaiese et al. 2018). In a quick search on PubMed, we noticed that the first papers on the subject of microalgae in agriculture were published in 2009 and currently less than a hundred articles can be found in this area.

Research results translate into innovative technologies once they reach the field and their commercial use begins. Potential inventions often become patent applications to ensure the protection of industrial innovation. Therefore, patent database analysis is a valuable approach to identify the developments in a particular field, mainly from the market point of view and a useful tool to forecast future trends and plan strategies for research development (Jara et al. 2016; Alba et al. 2021). Such analysis is also a useful tool to examine the flow of information from science to technology especially for emerging technologies (Sastry and Rashmi 2010).

Patents can be used as reference for the scientific community due to the level of detail of technical information, open access database, accessibility and availability of patent submissions and the publication timeline (Talebi and Tabatabaei 2018). Therefore, the present work analyzed the current status of microalgae patents in agriculture with the aim to provide an analysis of the patent behavior in this field. Patent analysis included: (i) bibliographic analysis and (ii) the technological analysis of microalgae products and their potential applications in agriculture.

## Database patent search and data analysis

**Fig. 1:**
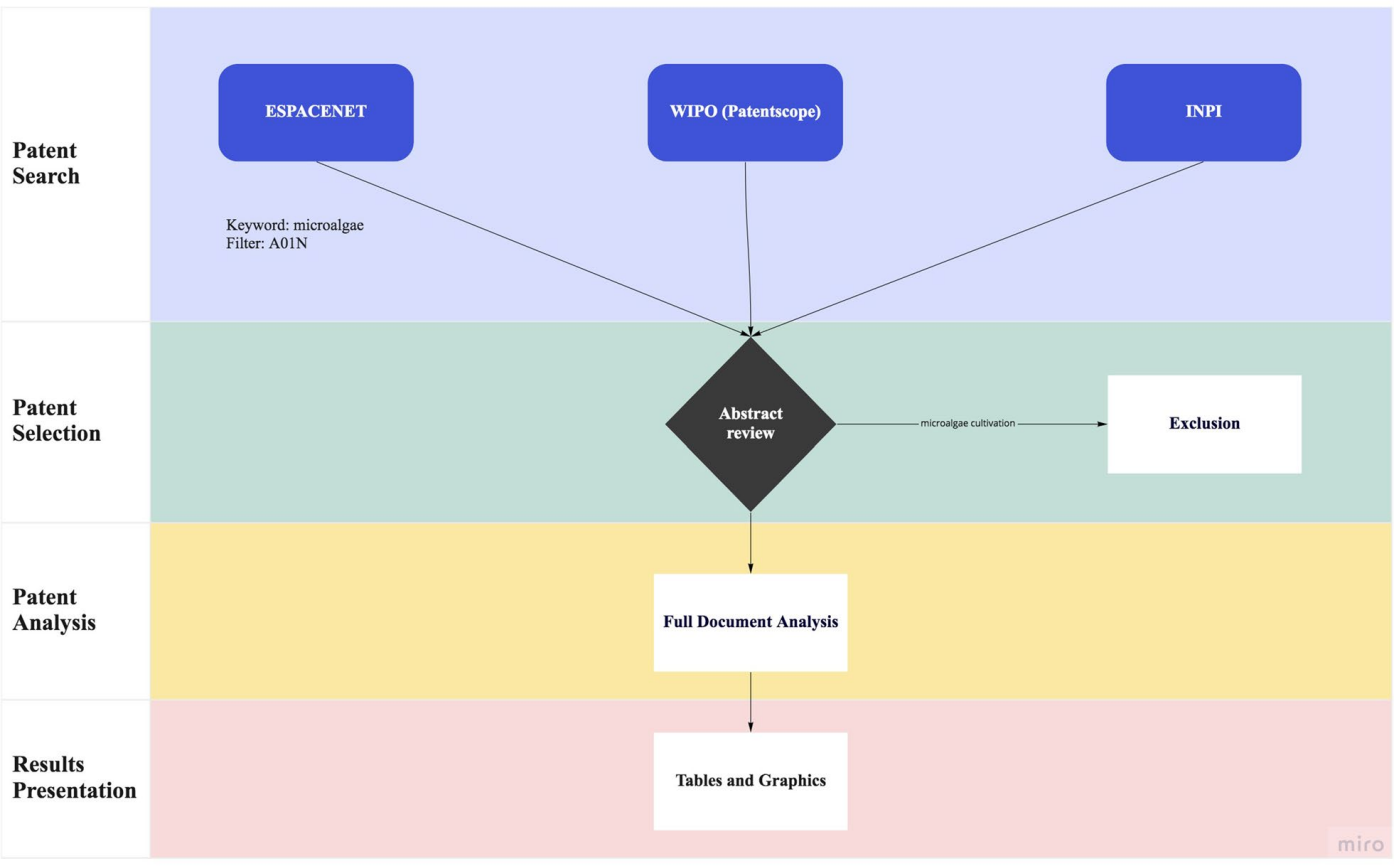
Diagram with the steps of the patent database search and data analysis

To start our analysis on microalgae agricultural patents, the search was applied to three different databases, such as Espacenet (European Patent Office searching engine), Patenscope (World Intellectual Property Organization database) and INPI (Brazilian National Institute for Industrial Property) from August 2020 to December 2020. The search was narrowed using the keyword “microalgae” and applying the filter “A01N” at the IPC main groups/IPC code. In the INPI database, the keyword used was “microalga” (Brazilian word for microalgae) and no filter was applied during the search (Fig. 1).

The International Patent Classification (IPC) is a hierarchical system used to classify patent documents according to different areas of technology, using letters and numbers as codes. The symbol A01 covers all patents related to agriculture, forestry, animal husbandry, hunting, trapping and fishing. The code “A01N” includes patents related to products such as biocides, pesticides, herbicides and plant growth regulators (WIPO 2020a, c).

During the search, 669 patents were found and only 132 patents were selected based on patent abstracts related to microalgal protocols or products with applications in agriculture. Patents related to microalgae cultivation were excluded because this subject does not meet our research scope. The documents were examined in full and analyzed according to patent title, office, application number, publication number, publication date, grant number, grant date, patent status, ICP code, country, applicants, inventors, abstract, patent purpose, target audience and microalgal species.

All selected patents were used for analyzing the timeline of patent applications, geographical distribution, and patent status. On the other hand, redundant patents present in more than one databases were excluded from the analysis of area of use and patent description to avoid result misinterpretation. Only one patent was considered if the same patent was published in more than one office.

Table 1 shows the number of patents found in each database and the number of selected patents after database and office redundancy exclusion. This filtering step is required because the same patent may be found in more than one database and can be registered in several offices around the world. Only 47 unique patents correspond to research using microalgae as an agricultural product (Table 1).

**Table 1:** Total number of patents related to microalgae in agriculture found in each database and the number of patents selected after each analysis

Databases	Total number of patents found	Number of patents selected	Number of patents selected	Number of patents selected
		(after abstract review)	(after database redundancy exclusion)	(after office redundancy exclusion)
Espacenet	526	94	–	–
WIPO	110	37	–	–
INPI	33	1	–	–
Total	669	132	125	47

## Historical perspective of microalgae patents in agriculture

**Fig. 2:**
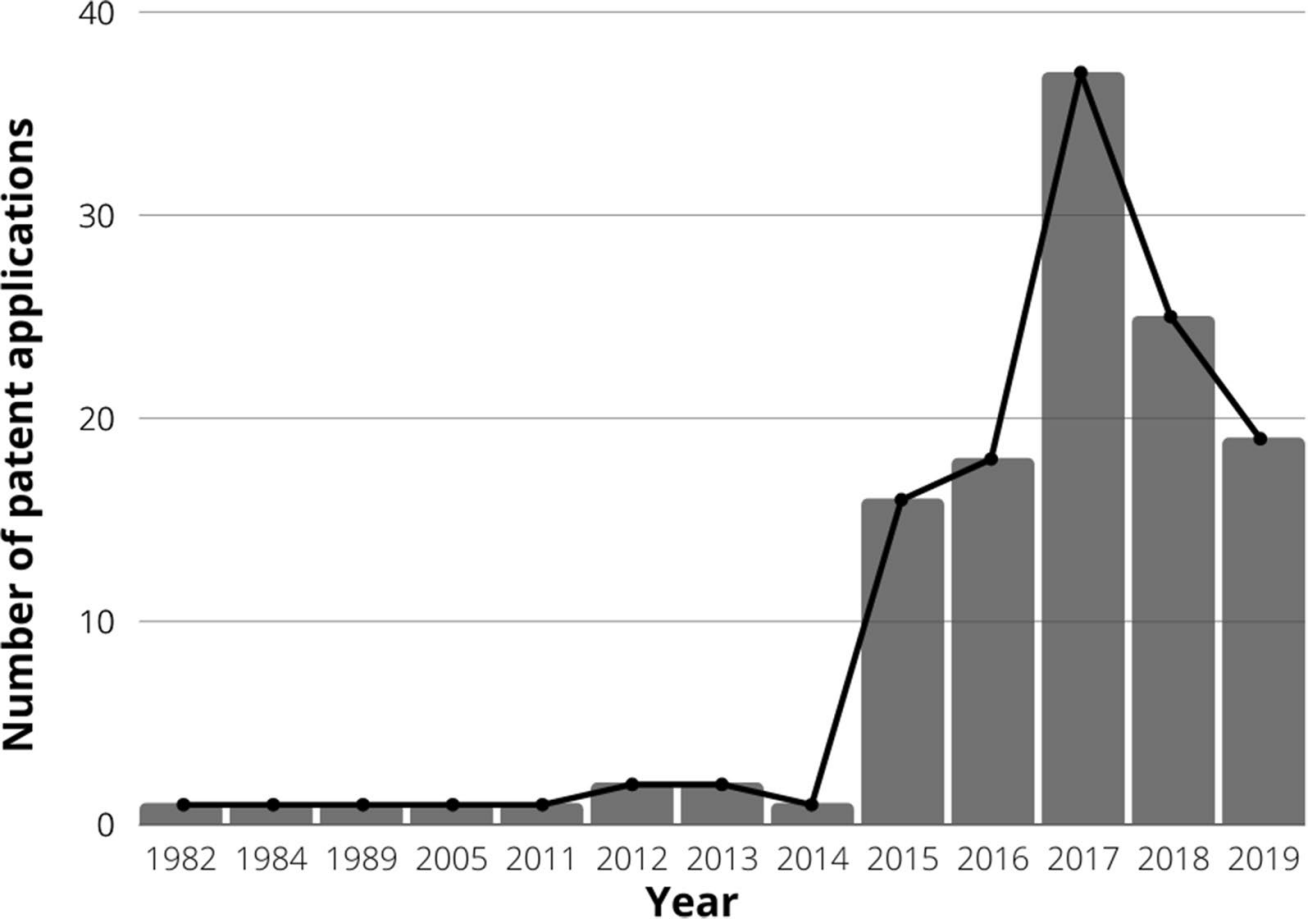
Application activity timeline of patents related to microalgae in agriculture per year

Figure 2 represents a historical perspective of microalgae in agriculture with 132 selected patents over the years (before redundancy exclusion). The first patent was registered in 1982 in Japan and described the use of microalga *Chlorella* extract as a plant growth promoter due to its effect on hormone biosynthesis as auxin and gibberellin (Naohiko 1982). Another two Japanese patents were registered in the 80 s describing similar benefits on seed germination and fruit ripening (Maeda 1984; Hishimuna et al. 1989). After that, no patent was registered for 15 years. From 2005 to 2014, only seven patents were found. The scenario changed from 2015 when the number of patent applications showed a sharp increase. (Fig. 2).

In 2016, the World Intellectual Property Organization (WIPO) published a document called “Patent landscape report on microalgae-related technologies.” This document reported an increase in microalgae patent activity between 2008 and 2013 due to the emergence of 3rd generation biofuel based on microalgae. However, as we can see in Fig. 2, patent applications related to microalgae in agriculture started to increase in 2015.

In general, the overall number of patents has been increasing. According to the European Patent Office (EPO) statistics, in 2019, the office received more than 180,000 applications, a new record. At WIPO, patent filings worldwide declined by 3% in 2019, however it was the first decline in patent applications since 2009. The increasing use of patents to protect inventions is closely related to a recent rise in the number of patent processes and the performance of the economy (OECD 2004).

The increase in microalgae patents in the agricultural area indicates a market shift towards organic food products and increasing costumer concern for sustainable agriculture. The consumption of organic foods has rapidly expanded over the last two decades, therefore the areas used for organic farming are also increasing. According to Willer et al. (2018), organic agricultural areas expanded by 7.5 million hectares or 15% in 2016. In 2017, the area for organic farming reached 69.3 million hectares (Ostapenko et al. 2020). In this scenario, the need for effective biological products grows at the same rate in order to meet this specific market demand in different countries.

## Geographical distribution of microalgae patents in agriculture

**Fig. 3:**
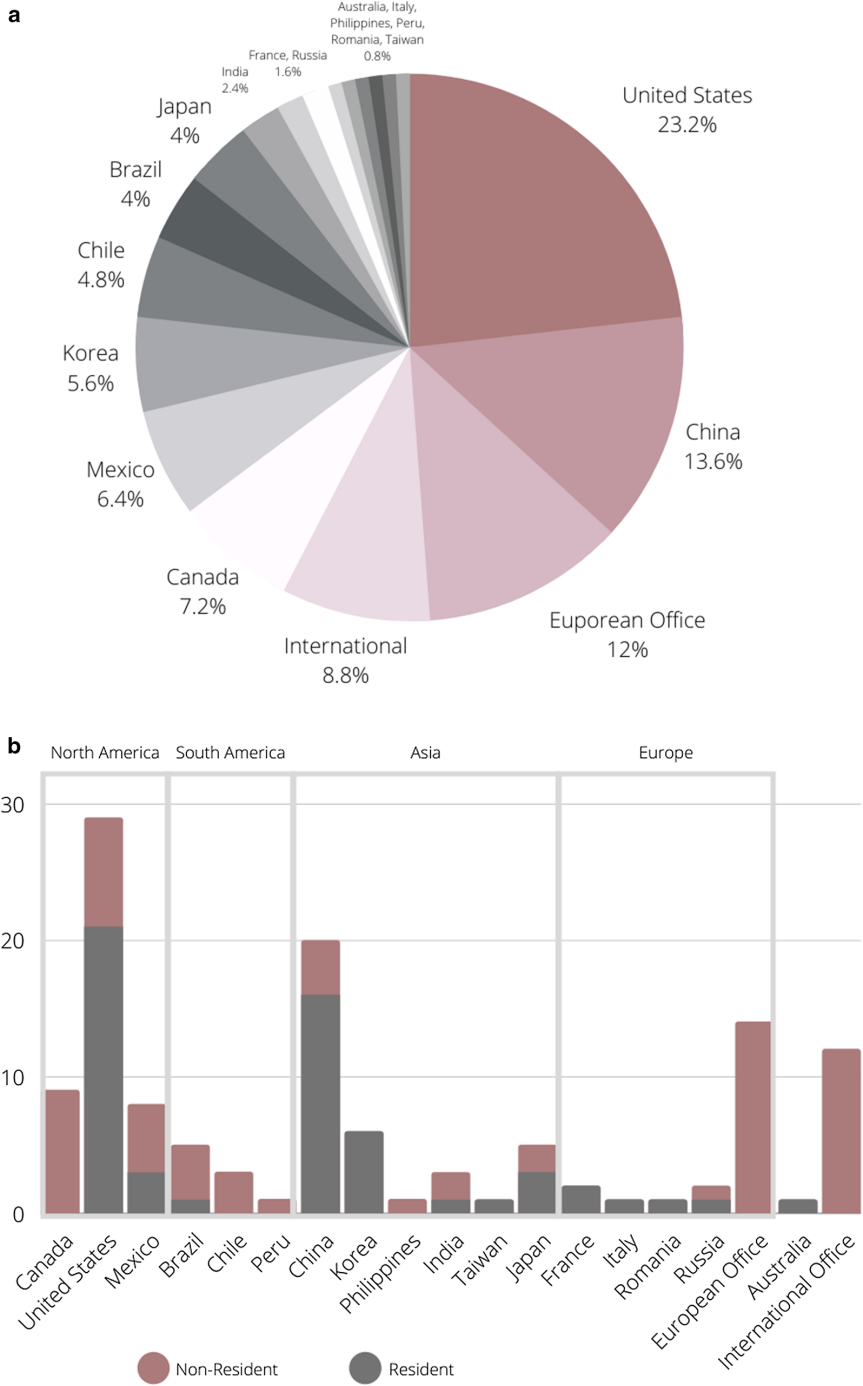
**a** Distribution of patent applications related to microalgae in agriculture according to patent offices around the world. **b.** The link between resident and non-resident patents related to microalgae in agriculture

Figure 3a shows the office distribution of 132 selected patents. The patents were submitted to 19 different offices. However half of the patents were registered in three main offices: The United States Office, China Office and European Office. Figure 3b shows the link between resident and non-resident patents. The term "resident" is used for filings made by applicants in their home office/country. Non-resident patents are applications filed with a patent office of a given country by an applicant residing in another country (WIPO 2021). United States, China, Korea and Japan have a higher number of resident patents. On the other hand, countries from South America and Canada showed a higher number of non-resident applications.

These results back up the fact that China and European countries contributed meaningly to the global organic farming growth, where the area grew from 0.67 to 1 million hectares in 2016 (Willer et al. 2018). In addition, organic food consumption is mainly concentrated in North America and Europe corresponding to 90% of sales. In contrast, regions like Latin America accounts for only 1% of this market (Willer and Lernoud 2018; Molinillo et al. 2020).

Even though South America has a small market for organic food, this continent has important agricultural countries such as Brazil, which is gradually developing interest for sustainable agriculture, explaining non-resident patent applications in Brazil, Chile and Peru (Fig. 3b). In Fig. 3b, we also observed that Canada did not develop any patents, however the nine patents submitted in this country revealed an interest in using microalgae-based products in Canadian agriculture. The same pattern was observed in Mexico. The main agricultural countries (China, United States, European countries, Brazil, Russia, India, Mexico and Japan) are represented in Fig. 3b, showing that microalgae-based products in agriculture are growing worldwide.

It is important to mention that European countries, United States and China lead not only in microalgae-based agricultural products but also in the overall number of patent applications (technology in all areas). In 2019, about half of all patent applications at the European Patent Office came from European companies. American firms corresponded to 25% of all applications followed by companies from Japan, China and South Korea (EPO 2019). WIPO showed a similar scenario in 2019. More than 84% of all patent filings in 2019 took place in the IP offices of China, the U.S., Japan, the Republic of Korea and the EPO. This global scenario showed that China, US and Europe are an attractive technology market for different areas, including sustainable agriculture.

## Area of expertise of microalgae patent applications

In Table 2, the patents were classified according to their area of expertise. After reading all material, the most prominent area was chosen to classify each patent document. The main agricultural areas found were Plant Growth, Fertilizers, Resistance Elicitors, Plant Health, Weed Management and Post-harvest.

**Table 2:** Number of patents* related to microalgae in agriculture according to application and area of use

Category	Division	Sub-division		Number of patents* submitted	Number of patents (no redundancy)
Agriculture	Plant growth	Growth promoter		46	17
	Biofertilizers	Biofertilizers		16	8
	Resistance elicitors	Resistance elicitors		24	6
	Plant health	Seed coating		7	1
		Fungicide/Bactericide		21	6
		Nematode		2	1
		Insects		2	1
	Weed management	Lawn protectors		4	4
	Post-harvest	Fruit bruise		2	1
		Fruit sweetness		1	1
Total				125	46

^*^ patents related to microalgal protocols or products used in agriculture

The Plant Growth category includes plant growth promoter products. The patents included in this category described the use of a microalgae extract/filtrate or microalgal biomass to increase seed germination rates and accelerate the emergence on *Fabaceae*, *Hydrangeaceae* and *Solanaceae* plants (Shinde et al. 2016; Hishinuma et al. 1989; Carney et al. 2019). Other patents described benefits of microalgae products on soil and overall plant improvement such as plant growth rate, plant length, flowering growth and yield (Cheng et al. 2017; Shinde et al. 2017; Florin et al. 2012). One patent described the use of microalgae extracts in stimulating hormone biosynthesis as auxin and gibberellin, enhancing plant development (Naohiko 1982). Phytohormones are essential compounds for plant development and exogenous supplementation of plant hormones in agriculture is a practice used to enhance crop yield (Egamberdieva et al. 2017, 2015). Many microalgae lineages are reported to produce or secrete phytohormones including auxin, abscisic acid (ABA), cytokinin (CK), ethylene (ET), and gibberellins (GAs) (Lu and Xu 2015). This Plant Growth area has the largest number of patents registered so far.

In the Plant Health category we included all patents describing microalgae products that efficiently reduce plant disease, infection, disease incidence and severity and insect damage. Experiments describing these products showed effects against fungi, oomycetes, bacteria, nematodes and insects, such as *Fusarium* sp., *Botrytis* sp., *Macrophomina* sp., *Rhizoctonia solani*, *Sclerotinia sclerotiorum*, *Verticillium* sp. (Shinde et al. 2017). Recent studies described the antifungal potential and antimycotoxigenic effect of microalgae phenolic extracts in wheat cultures in vitro (Scaglioni et al. 2019). A similar study revealed that microalgae phenolic extracts were also capable of inhibiting the production of the mycotoxin fumonisin in vitro more efficiently than tebuconazole (Scaglioni et al. 2018).

Terra et al. (2019) reviewed the ability of microalgae to synthesize nanoparticles, since they can convert toxic metals to nontoxic forms by chelation, incorporating the metals in successive chemical reactions and nanoparticle formation (Mahdieh et al. 2012). Terra et al. (2019) discussed the antimicrobial potential of silver nanoparticles synthesized by microalgae for pathogen control in agriculture and highlighted the advantages of combining the biosynthesis of the nanomaterial with the production of microalgal biomass. The microalgal biomass also exhibits antimicrobial properties and increases the antibacterial and antifungal properties of this technology.

Another category related to Plant Health is Resistance Elicitor. One invention claims that microalgae filtrate or biomass can increase plant immunity through the activation of salycilic acid and jasmonic acid pathways (Moo and Min 2019). Salicylic acid (SA) is a key plant hormone that mediates host responses against pathogens through the activation of plant defenses, especially systemic acquired resistance (SAR) (Kumar 2014). Jasmonic acid (JA) is another plant hormone responsible for the regulation of many physiological processes in plant growth and development, especially the mediation of plant responses to biotic and abiotic stresses (Ruan et al. 2019). Another patent in this category described the use of microalgae extracts in the induction of plant resistance. In this invention the microalgal extract had a high polysaccharide content, including chrysolaminarin, which has been described as an important plant resistance elicitor (Guido 2017). Studies with biochemical and metabolic markers showed that microalgal polysaccharides successfully improve enzyme activity linked to defense pathways in tomato plants, such as phenylalanine ammonia lyase (PAL), chitinase, 1,3-beta-glucanase and peroxidase (POX) (Stadnik and Freitas 2014; Rachidi et al. 2021). In addition, the same treatments with microalgae polysaccharides exhibited positive effects on chitinase, peroxidase, and polyphenols. Those metabolites are frequently studied in plant defense research and are known to correlate with the occurrence of induced resistance (Stadnik and Freitas 2014; Rachidi et al. 2021).

Biofertilizer is another important agricultural product derived from microalgal biomass. The benefits described in the patents are increased soil cation exchange capacity, soil nutrient availability, atmospheric nitrogen fixation and organic matter and essential microelement input. Consequently, the use of microalgae-based biofertilizers resulted in improved plant emergence and growth. Examples of beneficial use of microalgae dry mass as fertilizer were reported in different experiments with onion, maize, rice, wheat and other crops (Dineshkumar et al. 2018, 2019, 2020; Renuka et al. 2016; Guo et al. 2020). Besides the benefits to the plant, microalgae are renewable resources that produce biomass using the nutrients in wastewater, due to its short life span, high growth rate and high CO2 utilization efficiency. Therefore, microalgae could be simultaneously used for wastewater treatment and biofertilizer use (Hussain et al. 2021). However, other studies point out that the production of microalgal biomass demands technological improvements in order to be sustainable due to highenergy consumption during the process. Castro et al. (2020) and Souza et al. (2019) demonstrated, using life cycle analysis, that microalgae-based biofertilizer can result in more environmental damage, such as climate change, compared to chemical fertilizers. The reason is high electricity consumption for low microalgae productivity. The biomass cultivation, including the dewatering and drying processes, was the most critical step and needs improvements to make microalgae biofertilizers environmentally advantageous.

Another category of patents is Weed Management that included four Korean patents, mainly lawn protectors (Lee 2016; Wok 2018). These inventions consisted of low-salinity microalga-containing salt preparations for removing lawn weed. Due to the salt accumulation in soil the weeds are simply and effectively removed at low costs (Lee 2016). And finally, there is a category called Post-Harvest which describes two patents of the same microalgae product PhycoTerra® that showed promising results on the decrease of post-harvest fruit bruise and the increase of fruit sweetness when applied to plants (Carney and Michael 2017; Carney et al. 2018).

## Description of granted microalgae patents in agriculture

**Fig. 4:**
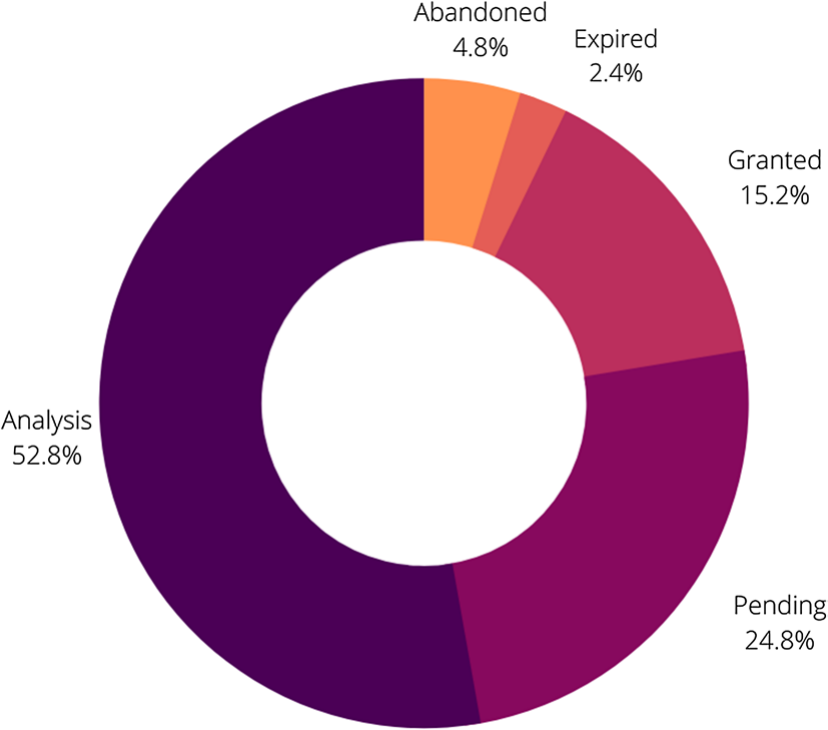
Statuses of patents related to microalgae in agriculture according to patent office analyses

For most of our analysis, all patents submitted in patent offices were taken into consideration. However, it is important to observe which of these patents were granted or denied. Figure 4 shows the distribution of 125 patents according to their status during the registration process. Most patents (53%) are being analyzed by registration offices around the world and 25% of these are in a pending situation, which means there is a lack of documentation that will delay the process. In addition, 7% of the patents were abandoned or expired and only 15% of patents (a total of 20) were granted (before redundancy exclusion).

After patent submission by the applicant, patent offices analyze applications and decide whether or not to grant patent rights. Patent applications should meet important criteria as standards of novelty, non-obviousness and industrial applicability are defined in national laws. Processing patents takes time and resources and examination processes differ across offices, which makes cross-country comparisons difficult (WIPO 2020a, c).

In Table 3, we described all granted patents found in our research. It is important to mention that among over one hundred patent applications selected for our study, only 10 patents were granted (after redundancy exclusion) and allowed to develop commercial products for growers. In patents selected for our analysis, the time from patent application to granting took an average of two years. Most of the granted patents were filed by private companies.

**Table 3:** Description of all microalgae patents related to agriculture granted by EPO and WIPO

References	Application Year	Grant Year	Publication number	Title	Product type	Microalgae species	Company’s name	Origin Country
Astakhov et al. (2005)	2005	2007	RU2005130370A	Method for biological protection of summer-planted potato from Colorado beetle	Insecticide	*Microcystis aeruginosa*	G NAUCHNOE UCHREZHDENIE VRII	Russia
Lee (2016)	2016	2017	KR2016019054A	Manufacturing method of microalgae-containing liquid salt for weeds elimination in lawn having low salt density	Herbicide	*Oscillatoria* sp.	Lee Se Yong	South Korea
Thiebeauld de la Crouee, O. and Thomas (2016)	2016	2018	FR1655263A	Cellular extract of one or more microalgae of the genus *Amphidinium* for its fungicide and/or bactericide activity on fungi, pathogenic oomycetes and/or bacteria of plants and culture seeds	Fungicide	*Amphidium carterae, Prymnesium parvum, Phaeodactylum tricornutum*	Immunirise	France
Carney and Michael (2017)	2017	2020	US201816010686A	Biomass compositions for decreasing bruising in fruit and methods therefor	Post-harvest	*Chlorella, Aurantiochytrium acetophilum, Galdieria, Scenedesmus, Haematococcus, Isochrysis, Spirulina*	Heliae Dev LLC	United States
Cheng et al. (2017)	2017	2020	TW106100901A	Soil improving composition and method for promoting plant growth which not only has rapid and long-acting nitrogen supplementation, but also inhibits the occurrence of pests and diseases	Growth promoter	*Chlorella* spp.	Tai Jian Biotech Co. LTD	Taiwan
Baek (2017)	2017	2018	KR101928514B1	Eco-friendly grass protectant using sea water	Lawn protector	*Chlororella* sp., *Paeodactylium* sp.	Purum Bio Co., LTD	South Korea
Moo and Min (2019)	2018	2019	KR20190090526A	Composition for improving immunity of plant comprising cultural filtrate of *Chlorella* sp.	Resistance elicitor	*Clorella* sp.	Korea Research Institute of Bioscience and Biotechnology	South Korea
Carney; Jauregui; Miller (2018)	2018	2020	US201916567597A	Compositions and methods for indirectly reducing incidence of fungal pathogen activity in plants	Fungicide	*Chlorella, Aurantiochytrium acetophilum, Galdieria, Scenedesmus, Haematococcus, Isochrysis, Spirulina*	Heliae Dev LLC	United States
Carney et al. (2018)	2018	2020	US20190142014A1	Biomass compositions for increasing sweetness of fruit and methods therefor	Post-harvest	*Chlorella, Aurantiochytrium acetophilum, Galdieria, Scenedesmus, Haematococcus, Isochrysis, Spirulina*	Heliae Dev LLC	United States
Mógor et al. (2019)	2019	2020	BR1020190065672	Microalgae enzymatic hydrolysis and product recovery based on free amino acids for agricultural use	Biofertilizer	*Arthrospira* sp. (*Spirulina* sp.)	Universidade Federal do Paraná	Brazil

These inventions use microalgal biomass or microalgae extract as seven different products: insecticides, herbicides, fungicides, biofertilizers, post-harvest products, growth promoters and resistance elicitors. Many patents overlap in their function and describe more than one benefit to the plant, mainly because most areas are correlated with each other. In summary, the application of microalgae stimulates soil microbial activity, facilitating nutrient availability and increasing soil fertility. As a result, the plant is healthier and grows more vigorously, enhancing crop yield and fruit quality (Renuka et al. 2018).

## Conclusion

In conclusion, our study showed that patent analysis was a useful tool for gathering recent information related to the use of microalgae-based products in agriculture, mainly related to the practical use of these products in the field. The interest in sustainable agriculture has increased the number of microalgae-related patents in the area of plant growth, plant health, resistance elicitors and biofertilizers. Even though research is being carried out around the world, there are still only a few granted patents that are available on the market, pointing out that there is room for development and improvement of microalgae use in the agricultural sector.

## Data Availability

The datasets that support the findings of this study were derived from the following resources available in the public domain: WIPO (https://patentscope.wipo.int/search/en/search.jsf), European Patent Office Espacenet (https://worldwide.espacenet.com/) and INPI (https://www.gov.br/inpi/pt-br/servicos/patentes).

## Abbreviations

EPO: European Patent Office
WIPO: World Intellectual Property Organization
INPI: Instituto Nacional de Propriedade Intelectual
IPC: International Patent Classification
IP: Intellectual Property
